# Impact of Preoperative Weight Loss on Prognosis in Patients with Pancreatic Cancer

**DOI:** 10.3390/biomedicines13071703

**Published:** 2025-07-12

**Authors:** Mariko Tsukagoshi, Kenichiro Araki, Norio Kubo, Takamichi Igarashi, Shunsuke Kawai, Kei Hagiwara, Kouki Hoshino, Takaomi Seki, Takayuki Okuyama, Ryosuke Fukushima, Takahiro Shoda, Ken Shirabe

**Affiliations:** Division of Hepatobiliary and Pancreatic Surgery, Department of General Surgical Science, Gunma University Graduate School of Medicine, 3-39-22 Showa-machi, Maebashi 371-8511, Gunma, Japan; marikot@gunma-u.ac.jp (M.T.); nkubo@gunma-u.ac.jp (N.K.); takamichi.iga@gunma-u.ac.jp (T.I.); moxjet-s59@yahoo.co.jp (S.K.); asikaika.rt@gmail.com (K.H.); h.kouki.915@gmail.com (K.H.); takaomi.seki@icloud.com (T.S.); okym.sprint.kicks100@gmail.com (T.O.); r.fukushima1025@gmail.com (R.F.); dokuganryusho@gmail.com (T.S.); kshirabe@gunma-u.ac.jp (K.S.)

**Keywords:** body weight, nutrition, outcome, pancreatic cancer, prognosis, surgery, weight loss

## Abstract

**Background/Objectives**: This study aimed to investigate the impact of preoperative weight loss on long-term postoperative survival and the significance of preoperative nutrition on perioperative weight change in patients with pancreatic cancer. **Methods**: Overall, 125 patients who underwent radical resection for invasive pancreatic ductal carcinoma were retrospectively analyzed. The preoperative weight loss rate (pre-%WL) from the initial visit to immediately before surgery was calculated. Patients were classified by pre-%WL into the weight-loss (≥6% loss) and weight-maintenance (<6% loss) groups. The association of pre-%WL with postoperative outcomes and long-term survival was assessed. We evaluated preoperative nutrition’s effect on perioperative weight change. **Results**: The study cohort included 91 (73%) and 34 (27%) patients with weight maintenance and weight loss, respectively. Specifically, the weight-loss group had a longer operative time (*p* = 0.025) and greater blood loss (*p* = 0.018) than the weight-maintenance group. Patients with weight loss had significantly poorer recurrence-free survival (RFS; 8.7 vs. 17.8 months, *p* = 0.004) and overall survival (OS; 18.1 vs. 45.2 months, *p* = 0.002) than those with weight maintenance. Multivariate analysis revealed weight loss as an independent prognostic indicator of poor RFS (hazard ratio = 2.07; *p* = 0.003) and OS (hazard ratio = 2.55; *p* = 0.0008). The presence or absence of preoperative nutritional therapy was not correlated with the pre-%WL but was associated with postoperative (by the time of discharge) weight loss rate (median weight change rate: −2.9% vs. −5.6%, *p* = 0.001). **Conclusions**: Preoperative weight loss ≥ 6% was associated with poor RFS and OS in patients with pancreatic cancer. Although preoperative nutritional therapy did not suppress preoperative weight loss, it suppressed postoperative weight loss.

## 1. Introduction

Pancreatic cancer is one of the most lethal malignancies, with a low 5-year relative survival rate of 11% [1]. Its incidence continues to rise, and it currently ranks as the fourth leading cause of cancer-related deaths worldwide [1,2]. Surgical resection remains the only potentially curative treatment option, and the number of pancreatic surgeries has shown a slight upward trend over the past 10 years.

Weight loss is common with the diagnosis of pancreatic cancer. Over 80% of patients with pancreatic cancer experience weight loss at the time of diagnosis, and approximately 40% lose > 10% of their initial body weight within 6 months of diagnosis [3,4,5]. Weight loss in patients with pancreatic cancer has predictive and prognostic implications [6]. Specifically, weight loss correlates with increased postoperative complications, longer hospital stays, mortality, and a reduced response to chemotherapy, as well as shorter progression-free survival (PFS) and overall survival (OS) [6,7,8]. Postoperative weight loss has also been reported as a factor affecting the continuity of adjuvant chemotherapy and a prognostic indicator of survival after pancreatectomy [9,10,11]. Despite these facts, weight loss during the perioperative period for pancreatic cancer has rarely been analyzed, and no evidence exists regarding specific interventions for its prevention.

Therefore, this study aimed to investigate the effect of preoperative weight loss on short- and long-term postoperative outcomes and that of preoperative nutrition on perioperative weight change in patients with pancreatic cancer.

## 2. Materials and Methods

### 2.1. Patient Selection

Overall, 125 patients who underwent radical resection for invasive pancreatic ductal carcinoma (PDAC) between January 2016 and December 2022 in the Department of Hepatobiliary and Pancreatic Surgery at Gunma University Hospital were retrospectively analyzed. The Ethics Committee of the Gunma University Hospital (approval number: HS2021-190) approved the study’s protocol. Informed consent was obtained from all participants, and all clinical samples were used in accordance with institutional guidelines and the principles of the Declaration of Helsinki.

### 2.2. Treatment and Data Collection

Baseline clinical and demographic characteristics, as well as treatment-related details of all patients, were obtained from medical records. Surgical procedures were performed according to institutional policies and recommendations from the institutional cancer board. The Clavien–Dindo classification was used to evaluate postoperative complications [12]. Complications requiring surgical intervention during the first 30-day preoperatively were defined as Clavien–Dindo grade III or higher. Resected tumors were classified according to the TNM staging system of the International Union Against Cancer.

### 2.3. Neoadjuvant Therapy, Postoperative Adjuvant Therapy, and Postoperative Follow-Up

Patients with borderline resectable pancreatic cancer were administered neoadjuvant therapy using the following regimen: nab-paclitaxel plus gemcitabine therapy, modified FOLFIRINOX, or S-1. In our institution, neoadjuvant therapy of gemcitabine plus S-1 has been used for patients with resectable pancreatic cancer since April 2020. All patients were treated with postoperative adjuvant therapy (S-1) except for those in poor general condition or those who declined treatment.

Recurrence-free survival (RFS) was defined as the period from the date of surgery until that of either recurrence or all-cause mortality. OS was defined as the period from the date of surgery to that of all-cause mortality.

### 2.4. Preoperative Changes in Body Weight and Nutrition Therapy

We examined body weight changes during the perioperative period. Patients’ body weight history was obtained over the following time frames: at the initial visit, immediately before surgery, and at discharge. The median duration between the initial visit and immediately before surgery (preoperative interval period) was 34 days. We defined preoperative weight loss rate (pre-%WL) as the percentage calculated by the following formula: [(weight immediately before surgery—weight at initial visit)/weight at initial visit] × 100. The postoperative weight loss rate (post-%WL) was defined as the percentage calculated by the following formula: [(weight at discharge − immediately before surgery)/weight immediately before surgery] × 100.

Depending on the individual case, nutritional supplements containing branched-chain amino acids (BCAAs) (Rehadays^®^, Otsuka Pharmaceutical Factory, Inc., Naruto, Japan and/or Mei Protein^®^, Meiji Holdings Co., Ltd., Tokyo, Japan) were administered 2 packs per day as preoperative nutritional therapy. Patients who underwent nutritional intervention for ≥7 days preoperatively were defined as the nutritional therapy group.

### 2.5. Statistical Analysis

Categorical variables were assessed using the chi-square or Fisher’s exact test, as appropriate. The Mann–Whitney *U* test was used to analyze continuous variables. Survival curves were estimated using the Kaplan–Meier method, and the log-rank test was used to analyze differences between the curves. Univariate and multivariate analyses of prognostic factors were calculated using the Cox proportional hazards model. Statistical significance was considered at the *p* < 0.05. All statistical analyses were performed using the JMP Pro 18 statistical software program (SAS Institute, Cary, NC, USA).

## 3. Results

### 3.1. Patients’ Characteristics in the Two Groups Classified by Pre-%WL

The receiver operating characteristic curve (ROC) was plotted to determine the optimum pre-%WL for predicting postoperative survival in patients with pancreatic cancer. Additionally, the best cutoff value of the pre-%WL for survival was −6.4 (area under the curve = 0.59). Based on this result, patients were classified into the weight-loss (≥6% loss) and weight-maintenance (<6% loss) groups.

The study cohort included 34 (27%) and 91 (73%) patients with weight loss and weight maintenance, respectively. Table 1 presents a comparison of the clinical characteristics between the two groups classified by the pre-%WL. No statistically significant associations were observed between weight loss and age, sex, body mass index, resectability, preoperative interval period, and preoperative chemotherapy. Serum albumin level was lower, and C-reactive protein (CRP) was higher in the weight-loss group than in the weight-maintenance group (*p* < 0.001 and *p* = 0.008, respectively). Regarding tumor-related factors, only DUPAN-2 was higher in the weight-loss group than in the weight-maintenance group (*p* = 0.001). No difference was found in the tumor size, the presence or absence of lymph node metastasis, or the rate of preoperative nutritional therapy between the two groups.

### 3.2. Comparison of Pre-%WL and Perioperative Outcomes

Table 2 summarizes the perioperative features of patients in the weight-maintenance and weight-loss groups. No difference in operative procedures was found between the two groups. The weight-loss group had a longer operative time (526 vs. 446 min, *p* = 0.025) and greater blood loss (422 vs. 308 mL, *p* = 0.018) than the weight-maintenance group. No significant differences in the length of postoperative hospital stay and Clavien–Dindo grade III or higher complications were observed between the two groups. The percentage of patients who completed adjuvant S-1 therapy was 49% (*n* = 45) and 44% (*n* = 15) in the weight-maintenance and weight-loss groups, respectively, with no significant difference (*p* = 0.689).

### 3.3. Correlation Between Pre-%WL and Long-Term Postoperative Outcomes

**Figure 1:**
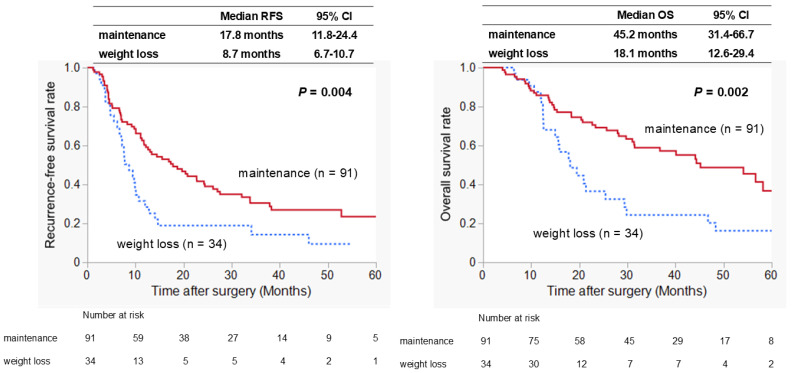
Comparison of long-term postoperative outcomes by preoperative weight loss. Patients with weight loss had significantly poorer recurrence-free survival (*p* = 0.004) and overall survival (*p* = 0.002) than those with weight maintenance. CI, confidence interval; OS, overall survival; RFS, recurrence-free survival.

Figure 1 shows the prognostic significance of patients in the weight-loss and weight-maintenance groups. Patients with weight loss ≥ 6% had significantly poorer RFS than those with weight maintenance (*p* = 0.004). Median RFS time was 8.7 and 17.8 months in patients with weight loss and those with weight maintenance, respectively. Regarding the OS rate, patients with weight loss had significantly poorer OS than those with weight maintenance (median survival time: 18.1 vs. 45.2 months, *p* = 0.002).

### 3.4. Prognostic Factors Associated with RFS and OS

Univariate and multivariate analyses were performed to analyze factors influencing RFS in all patients (Table 3). Univariate analyses revealed weight loss, resectability (borderline resectable), CA19-9 ≥ 150 U/mL, tumor size ≥ 30 mm, positive lymph node, pancreatoduodenectomy (PD) or total pancreatectomy (TP), and S-1 adjuvant therapy < 6 months as significant factors for reduced RFS. Multivariate analysis revealed weight loss (Hazard ratio [HR] = 2.07; 95% confidence interval [CI]: 1.28–3.34; *p* = 0.003), borderline resectable, PD or TP, and S-1 adjuvant therapy < 6 months as independent prognostic indicators of poor RFS.

Table 4 presents the results of the univariate and multivariate analyses for predicting OS. Univariate analysis revealed weight loss, tumor size ≥ 30 mm, PD or TP, and S-1 adjuvant therapy < 6 months as significant factors for reduced OS. The independent predictive factor detected by multivariate analysis was weight loss (HR = 2.55; 95% CI: 1.48–4.40; *p* = 0.0008) and S-1 adjuvant therapy < 6 months (HR = 3.89; 95% CI: 2.20–6.89; *p* < 0.0001).

### 3.5. Effect of Preoperative Nutritional Therapy on Weight Loss Group

**Figure 2:**
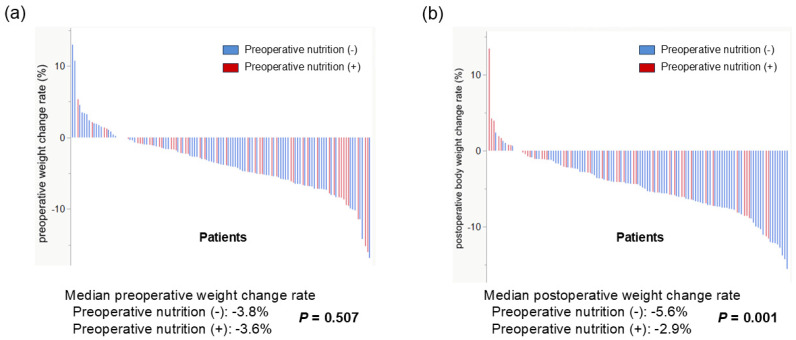
Perioperative body weight changes according to preoperative nutritional therapy. (**a**) No significant difference in preoperative weight loss rate was found between the groups with and without nutritional therapy (*p* = 0.507). (**b**) A significant difference in postoperative weight loss rate was found between the groups with and without nutritional therapy (median weight change rate: −2.9% vs. −5.6%, *p* = 0.001).

Based on our analysis showing that the pre-%WL was significantly associated with postoperative prognosis in patients with pancreatic cancer, we subsequently investigated the effect of preoperative nutritional therapy on perioperative weight change. Figure 2a shows the pre-%WL. The median weight change rate was −3.6% and −3.8% in the groups with and without nutritional therapy, respectively. No significant difference was found between the two groups (*p* = 0.507). Figure 2b shows the post-%WL. A significant difference in the postoperative weight loss rate was found between the groups with and without nutritional therapy (median weight change rate: −2.9% vs. −5.6%, *p* = 0.001).

## 4. Discussion

This study revealed the association between the pre-%WL and postoperative prognosis in patients with pancreatic cancer. Weight loss ≥ 6% was an independent postoperative prognostic factor of poor RFS and OS. Preoperative nutritional therapy has been shown to suppress postoperative weight loss. These findings suggest that preoperative weight loss could be a screening tool for predicting postoperative prognosis and is important in determining treatment options such as suitability for surgery and nutritional therapy in patients with pancreatic cancer.

Weight loss is highly prevalent among patients with pancreatic cancer. Progressive weight loss is one of the most significant and difficult-to-treat findings for patients with pancreatic cancer. Weight loss at the time of diagnosis in patients with pancreatic cancer has been previously reported. Weight loss > 5%, based on current criteria for cancer cachexia at cancer diagnosis, was present in 71.5% of patients with PDAC and was independently associated with higher baseline body mass index, longer symptom duration, and increased tumor size [13]. While the percentage of weight loss was a risk factor for higher levels of malnutrition, it was not associated with OS or tumor stage [3,4,14]. Nemer et al. [13] reported that patients with weight loss (>5%) at cancer diagnosis had a similar median survival time compared to those without significant weight loss (9.5 vs. 12.6 months, respectively, *p* = 0.40). However, weight loss was independently associated with worse survival when the threshold was raised to >10%.

In this study, we focused on weight loss from the time of initial visit to immediately before surgery and showed that weight loss of ≥6% was significantly associated with increased operative time and blood loss, as well as poor long-term prognosis. Several studies have investigated the association of perioperative weight loss with short- and long-term postoperative outcomes. Nishikawa et al. [15] found that rapid preoperative body weight loss ≥ 1.34% per month was an independent predictor of worse survival of patients with PDAC. A study by Kuwabara et al. [16] focused on weight loss from the time of admission to that of discharge. Regarding the duration of postoperative hospitalization, the group with a %WL of >10% exhibited a significantly worse OS than the group with a %WL of ≤10%. However, the difference was not significant for RFS. In that study, an operative time of >450 min and postoperative complications were independent risk factors for a %WL of ≥10%. Weight loss in patients with pancreatic cancer is progressive even after diagnosis, and short-term weight loss may be related to postoperative prognosis.

Weight loss during neoadjuvant chemotherapy (NAC) is reportedly associated with perioperative outcomes. From the National Surgical Quality Improvement Program database, of the 5590 patients with pancreatic adenocarcinoma who received NAC, 913 (16%) experienced significant weight loss, defined as at least 10% body weight loss in the 6 months preoperatively [17]. Significant weight loss was an independent preoperative predictor of a postoperative complication [17]. Patients who had significant weight loss were more likely to undergo unplanned intubation postoperatively, have postoperative ventilator need >48 h, have postoperative septic shock, and undergo reoperation. However, no differences were observed for pancreatic fistula, readmission rates, or 30-day mortality [17]. Other reports have shown that weight loss was not associated with pathologic or perioperative outcomes [18]. Our study included 28% of cases where preoperative chemotherapy was administered, although no significant difference was found between weight loss and the presence or absence of preoperative chemotherapy. No significant differences in perioperative outcomes were found between the weight-loss or weight-maintenance group. Regarding prognosis, weight loss during NAC reportedly does not predict poor prognosis, while weight gain (≥5%) has the potential to improve RFS [18]. Therefore, monitoring weight changes during pancreatic cancer treatment is important.

Several studies have highlighted the role of DUPAN-2 in predicting survival outcomes in patients with pancreatic cancer. A meta-analysis of 22 studies involving 4765 patients with pancreatic cancer [19] found that elevated levels of DUPAN-2 were linked to vascular invasion, highlighting its role in pancreatic cancer progression. Conversely, normalized DUPAN- 2 levels were associated with higher resectability and lower N-stage, underscoring its prognostic importance. Our present study showed the correlation between pre-%WL and DUPAN-2. Elevated preoperative DUPAN-2 levels were suggested to be an independent predictor of early recurrence in patients with pancreatic cancer [20]. Pre-%WL may reflect the pathological aggressiveness of pancreatic cancer, similarly to DUPAN-2, and may be associated with poor RFS. However, these are only our hypotheses, and further investigation is required to identify the processes through which preoperative weight loss contributes to prognosis.

We also assessed the impact of preoperative nutrition therapy. To date, no treatment has been shown to be beneficial in preventing perioperative weight loss. Weight loss is caused by an imbalance between energy intake and expenditure and has different causes. It can be caused by a decrease in BCAA levels and an increased resting metabolic rate due to the inflammatory response [21]. Our previous study revealed inadequate preoperative nutritional support and rehabilitation therapy as an independent risk factor for pancreatic fistula post-PD in patients with skeletal muscle loss [22]. In the present study, while preoperative nutritional therapy, including BCAAs, was ineffective in reducing preoperative weight loss, it showed the potential to suppress postoperative weight loss.

The median weight loss over the first year after pancreatectomy was reportedly −6.6% [9]. Previous studies reported that body weight loss was an important factor affecting the continuity of adjuvant chemotherapy. A previous study revealed postoperative body weight loss ≥ 10% as the most important factor affecting the continuity of adjuvant chemotherapy [11]. In the present study, multivariate analysis detected S-1 adjuvant therapy < 6 months as another independent predictive factor. Notably, preoperative nutritional therapy can be used to prevent postoperative weight loss, potentially leading to the completion of adjuvant therapy and even extending prognosis.

This study has some limitations. First, this was a single-center retrospective observational study with a small sample size. Second, since body weight measurement was performed at the initial visit, variations in the period from diagnosis among patients could have occurred. Third, the duration of preoperative or postoperative chemotherapy, regimens used, and dose intensity varied between patients, potentially affecting long-term outcomes. Fourth, nutritional management varied depending on the attending physician, potentially influencing weight trends. Fifth, this study did not examine differences in molecular features between patients in the weight-maintenance and weight-loss groups. The molecular mechanism behind this study is considered an important topic for future investigation. Therefore, larger prospective studies are warranted to validate and refine these findings.

## 5. Conclusions

Preoperative weight loss ≥ 6% was correlated with increased operative time and blood loss and was significantly associated with poor RFS and OS in patients with pancreatic cancer. Preoperative administration of BCAAs may help suppress postoperative weight loss. These results suggest that preoperative weight loss is a simple prognostic predictor for patients with pancreatic cancer and is important in determining the appropriateness of surgery and guiding preoperative nutritional therapy.

## Figures and Tables

**Table 1:** Patient characteristics.

Variables	Weight Maintenance (*n* = 91)	Weight Loss (*n* = 34)	*p*-Value
Age (years)	73 (42–88)	73 (44–86)	0.818
Male sex	38 (42%)	20 (59%)	0.108
Body mass index (kg/m^2^)	21.0 (15.6–31.0)	20.9 (16.8–31.7)	0.689
Resectability			1.000
Resectable	81 (89%)	31 (91%)	
Borderline resectable	10 (11%)	3 (9%)	
Preoperative interval period (days)	33 (12–667)	34 (13–129)	0.615
Preoperative chemotherapy	25 (27%)	10 (29%)	0.826
Parameters			
Albumin (g/dL)	4.2 (3.2–4.8)	3.9 (2.3–4.7)	<0.001 *
Hemoglobin (g/dL)	13.2 (7.1–15.9)	12.4 (8.2–17.3)	0.069
Lymphocytes (/μL)	1540 (320–3410)	1430 (350–2550)	0.447
CRP (mg/dL)	0.07 (0.01–3.32)	0.15 (0.01–18.14)	0.008 *
Zn (µg/dL)	76 (56–131)	76 (35–117)	0.410
CA19-9 (U/mL)	69 (1–1292)	82 (1–22472)	0.739
DUPAN-2 (U/mL)	81 (25–11706)	294 (25–16000)	0.001 *
Span-1 (U/mL)	41.6 (10–1325)	55.6 (10–5115)	0.156
Tumor size (mm)	30 (0–610)	33 (18–162)	0.119
Positive lymph node	59 (65%)	24 (71%)	0.671
Preoperative nutritional therapy	30 (33%)	15 (44%)	0.297

Data are expressed as median (interquartile range) or number of patients (%). * *p* < 0.05. Abbreviations: CRP, C-reactive protein; Zn, Zinc.

**Table 2:** Comparison of perioperative outcomes by preoperative weight loss.

Variables	Weight Maintenance (*n* = 91)	Weight Loss (*n* = 34)	*p*-Value
Operative procedures			0.140
Pancreatoduodenectomy/Total pancreatectomy	55 (60%)	26 (76%)	
Distal pancreatectomy	36 (40%)	8 (24%)	
Operative time (min)	446 (188–776)	526 (211–730)	0.025 *
Blood loss (mL)	308 (10–2964)	422 (23–2148)	0.018 *
Postoperative hospitalization (days)	21 (10–82)	22 (10–62)	0.809
Complications (Clavien–Dindo grade ≥ III)	26 (29%)	6 (18%)	0.255
Adjuvant S-1 therapy completion	45 (49%)	15 (44%)	0.689

Data are expressed as median (interquartile range) or number of patients (%). * *p* < 0.05.

**Table 3:** Univariate and multivariate analyses of variables for recurrence-free survival.

Factor	Univariate Analysis			Multivariate Analysis		
	HR	95% CI	*p*-Value	HR	95% CI	*p*-Value
Weight loss	1.93	1.22–3.04	0.005 *	2.07	1.28–3.34	0.003 *
Female sex	1.05	0.69–1.59	0.829			
Borderline resectable	2.04	1.08–3.85	0.028 *	3.72	1.85–7.47	0.0002 *
Neoadjuvant chemotherapy	1.43	0.89–2.31	0.142			
Age ≥ 75 years	1.26	0.76–2.09	0.384			
CA19-9 ≥ 150 U/mL	1.61	1.03–2.50	0.035 *	1.51	0.96–2.35	0.072
Tumor size ≥ 30 mm	1.69	1.09–2.62	0.019 *	1.27	0.82–2.01	0.305
Positive lymph node	1.72	1.07–2.75	0.025 *	1.36	0.83–2.23	0.229
R1 resection	1.39	0.82–2.36	0.223			
PD, TP	2.14	1.32–3.45	0.001 *	2.00	1.18–3.41	0.012 *
Complications (Clavien–Dindo grade ≥ III)	0.93	0.57–1.53	0.785			
S-1 adjuvant therapy < 6 months	2.95	1.91–4.55	<0.0001 *	2.94	1.86–4.65	<0.0001 *

* *p* < 0.05. Abbreviations: HR, hazard ratio; CI, confidence interval; PD, pancreatoduodenectomy; TP, total pancreatectomy.

**Table 4:** Univariate and multivariate analyses of variables for overall survival.

Factor	Univariate Analysis			Multivariate Analysis		
	HR	95% CI	*p*-Value	HR	95% CI	*p*-Value
Weight loss	2.22	1.32–3.73	0.003 *	2.55	1.48–4.40	0.0008 *
Male sex	1.13	0.69–1.84	0.632			
Borderline resectable	1.48	0.73–2.99	0.280			
NAC	1.01	0.55–1.88	0.964			
Age ≥ 75 years	1.12	0.67–1.86	0.668			
CA19-9 ≥ 150 U/mL	0.98	0.57–1.67	0.933			
Tumor size ≥ 30 mm	1.78	1.05–3.02	0.033 *	1.32	0.76–2.30	0.325
Positive lymph node	1.70	0.97–2.96	0.064	1.51	0.84–2.71	0.171
R1 resection	1.47	0.81–2.66	0.207			
PD, TP	1.77	1.02–3.09	0.044 *	1.22	0.68–2.19	0.510
Complications (Clavien–Dindo grade ≥ III)	0.90	0.51–1.59	0.729			
S-1 adjuvant therapy < 6 months	3.90	2.26–6.74	<0.0001 *	3.89	2.20–6.89	<0.0001 *

* *p* < 0.05. Abbreviations: HR, hazard ratio; CI, confidence interval; PD, pancreatoduodenectomy; TP, total pancreatectomy.

## Data Availability

The data presented in this study are available on request from the corresponding author due to privacy and ethical reasons.

## Abbreviations

The following abbreviations are used in this manuscript:

ROC	Receiver operating characteristic curve
RFS	Recurrence-free survival
PDAC	Pancreatic ductal carcinoma
PFS	Progression-free survival
OS	Overall survival
CRP	C-reactive protein
PD	Pancreatoduodenectomy
TP	Total pancreatectomy
HR	Hazard ratio
CI	Confidence interval
NAC	Neoadjuvant chemotherapy
BCAA	Branched-chain amino acids
Pre-%WL	Preoperative weight loss rate
